# mPFC-rTMS for patients with insomnia disorder using resting-state functional magnetic resonance imaging: a protocol for a randomized controlled trial

**DOI:** 10.1186/s13063-022-06934-1

**Published:** 2022-12-12

**Authors:** Jingjing Sun, Guohai Li, Danwei Zhang, Kaimo Ding, Jun Zhu, Si Luo, Wenyue Xu, Zhoubing Wang

**Affiliations:** Zhenjiang Mental Health Center, No. 199 Tuanshan Road, Zhenjiang, Jiangsu China

**Keywords:** mPFC-rTMS, Insomnia disorder, fMRI

## Abstract

**Background:**

Insomnia is the most common sleep disorder. Repetitive transcranial magnetic stimulation (rTMS) is safe and effective for insomnia disorder (ID). Convergent evidence show that the medial prefrontal cortex (mPFC) may be involved in the regulation of sleep and awakening at the cortical level and may serve as a potential target of rTMS in the treatment of ID. The purpose of this clinical trial is to study the efficacy of mPFC-rTMS in the treatment ID and explore the neural mechanism using resting-state functional magnetic resonance imaging (fMRI).

**Methods and design:**

This will be a parallel-group randomized, patient- and assessor-blinded trial. The study will recruit 60 ID patients assigned to a real mPFC-rTMS group or a sham mPFC-rTMS group. The allocation ratio is 1:1, with 30 subjects in each group. Interventions will be administered five times per week over a 4-week period, with an 8-week follow-up period. All participants will undergo neuropsychological and fMRI evaluations. The primary outcome measure of this study is the change scores of the Pittsburgh Sleep Quality Index (PSQI). The secondary outcome measures include the fMRI measurements, the Hamilton Depression Scale (HAMD), the Hamilton Anxiety Scale (HAMA), a sleep diary, and a polysomnography. Assessment of all parameters will be performed at baseline, post-treatment, and during follow-up.

**Discussion:**

It is expected that the study results will provide strong evidence of the effectiveness and the neural mechanism by which mPFC-rTMS improves sleep quality in ID patients.

**Trial registration:**

Chinese Clinical Trials Register ChiCTR2100054154. Registered on 10 December 2021.

## Introduction

Insomnia is the most common sleep disorder [1]. Due to the increase in social pressure and aging population, the prevalence of insomnia will continue to increase [2]. A recent meta-analysis of the prevalence of insomnia disorder (ID) in the general population in China showed that the prevalence rate of ID is 15.0% [3]. The long-term existence of insomnia may be the cause of the huge social and economic burden of this disease and can lead to cognitive dysfunction, psychological abnormalities, and increased suicide tendency [4]. Therefore, early identification and early effective therapies for ID patients are especially important.

At present, the first-line therapies for insomnia recommended by sleep guidelines are cognitive behavioral therapy and hypnotic medication. However, many insomniacs have poor compliance with cognitive behavioral therapy in clinical practice, and long-term use of hypnotic medication also has side effects and increase suicide risks. In recent years, there have been an increasing number of novel physical methods to treat insomnia, such as repetitive transcranial magnetic stimulation (rTMS), which has emerged as a new option due to its low side effects.

As a neuromodulation technique, the common therapeutic target of rTMS is the dorsolateral prefrontal cortex (DLPFC). Many studies [5–7] applied rTMS (on right DLPFC, 1 Hz) for insomniacs, and the results indicated that rTMS can improve the sleep quality of insomniacs and the effect is better than hypnotic medication without side effects. These results suggest that rTMS is safe and effective for primary insomnia.

Huang et al. [8] choose the posterior parietal lobe as the stimulated position for insomnia patients with generalized anxiety. They showed that the anxiety and insomnia symptoms were improved significantly. However, some studies suggest that rTMS does not significantly improve insomnia symptoms in Parkinson’s disease [9]. A recent meta-analysis suggested that rTMS was effective in ID patients, while the placebo effect of sham stimulation was significant [10].

The aforementioned studies are focused on the right DLPFC or posterior parietal cortex with low-frequency rTMS. Although rTMS display a certain effect, but there are still some patients who cannot benefit from it. The current studies did not involve abnormal brain network of insomnia patients as a stimulation target; then in this study, we will explore the therapeutic effects and neural basis of another new stimulated target.

Insomnia is an abnormal sleep pattern. Two important neurotransmitter receptors (dopamine receptors and adenosine receptors) involved in sleep arousal are expressed in the nucleus accumbens (NAc) [11, 12]. In addition, the glutamate neurons of the thalamus paraventricular nucleus and the ventral tegmental area can control awakening by regulating the NAc [13, 14]. Therefore, the NAc may play an important role in regulating sleep and awakening. Lazarus et al. [15] proposed a network model of sleep-wake regulation, suggesting that adenosine and dopamine receptors in NAc regulate sleep-wake behavior through cortical activation, mainly involving the excitatory neural projection from the medial prefrontal cortex (mPFC) to NAc. In addition, mPFC can directly project excitatory neurons downward to the sleep-wake regulatory system such as tuberopapillary nucleus of hypothalamus, lateral hypothalamus, and locus blueleus. These results suggest that mPFC may be involved in the regulation of sleep and awakening at the cortical level and may serve as a potential target of rTMS in the treatment of chronic insomnia.

Two recent neuroimaging studies used functional magnetic resonance imaging (fMRI) to analyze the resting state functional connection (RSFC) of NAc in ID patients and revealed abnormal RSFC between NAc and mPFC which suggest the brain related to the severity of insomnia [16, 17]. Shao et al. [16] proposed an abnormal NAC-mPFC circuit in ID. In addition, the hypothalamus, a central role in sleep-wake regulation, was recently used as the seed for functional connection by fMRI, and the results showed that the bilateral hypothalamus and bilateral mPFC resting state functional connection were enhanced in ID patients and were positively correlated with sleep quality [18]. Moreover, neuroimaging revealed reduced gray matter density [19, 20], abnormal regional homogeneity (ReHo) [21], amplitude of low-frequency fluctuations (ALFF) [22], and reduced activation of mPFC [23, 24]. These studies suggest that mPFC may play an important role in the pathophysiological mechanism of insomnia.

Therefore, we hypothesized that 1-Hz rTMS stimulation over mPFC would improve insomnia symptoms of ID patients by altering mPFC seed functional connectivity in the group receiving real mPFC-rTMS group compared with the sham mPFC-rTMS group.

## Methods/design

### Objective

The study objectives are (1) to determine the efficacy of mPFC-rTMS for ID and (2) to elucidate the neural mechanisms for the effects of mPFC-rTMS on ID.

### Trial design

**Fig. 1:**
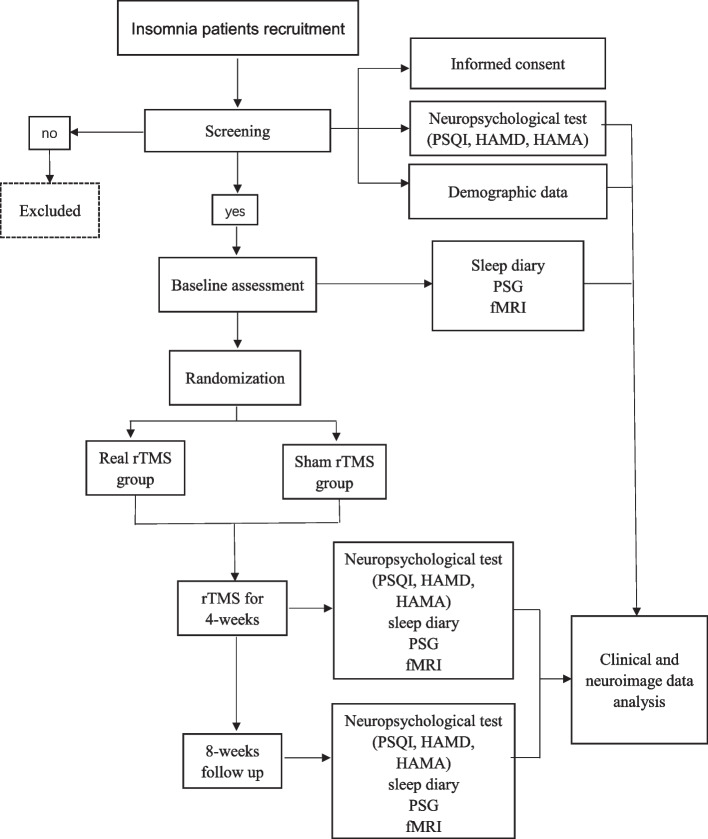
Flow chart of the study. PSQI, Pittsburgh Sleep Quality Index; HAMD, Hamilton Depression Scale; HAMA, Hamilton Anxiety Scale; PSG, polysomnography; fMRI, functional magnetic resonance imaging

This study is designed as a prospective parallel-group, patient- and assessor-blinded, randomized controlled single-center clinical trial with two parallel groups to detect the superiority of real mPFC-rTMS over the sham mPFC-rTMS. Digital randomization table will be performed as block randomization with a 1:1 allocation assigned to either a real mPFC-rTMS group or a sham mPFC-rTMS group. The neuropsychological measurements, a 1-week sleep diary, a 1-day polysomnography (PSG), and fMRI scans will be performed at baseline, post-treatment (immediately after completion of the 20-day treatment), and 8 weeks follow-up (8 weeks after the last session). The detailed flowchart is shown in Fig. 1. The trial will be conducted in accordance with the SPIRIT reporting guidelines [25]. Ethical approval has been granted by the ethics committee of Zhenjiang mental health center (202007), and the trial is registered at ChiCTR (CHiCTR2100054154).

### Sample size

This study aims to determine the efficacy and brain mechanism of mPFC-rTMS in ID patients. Based on preliminary experiments, the PSQI score significantly decreased by 4.43 ± 3.60 in the group treated with mPFC-rTMS compared to 1.30 ± 2.58 in the control group. Based on a power analysis, 26 patients per group were required to detect a significant difference (power = 0.9, *α* = 0.05, two-sided). Thus, considering a 15% dropout rate, we plan to recruit 30 patients per group to compensate. As to fMRI research, there is no known sample size calculation. However, for the exploratory study, 15 to 30 patients are adequate to test the null hypothesis [26].

### Participants and recruitment

We will post recruitment information in the hospital to recruit participants who report clinically significant insomnia. Prior to recruitment, the investigator will inform participants of the benefits, as well as possible risks (poor therapeutic effects and adverse events associated with rTMS) in the study. Informed consent will be obtained by investigators. All participants will sign informed consent forms before collecting data and randomization. Investigators will also sign the informed consent forms and record the informed consent process in the medical records.

Investigators will decide whether the participants meet the inclusion criteria based on the scores of PSQI, HAMD, and HAMA. The eligible participants will complete a baseline rs-fMRI, a 1-week sleep diary, and a 1-day polysomnography (PSG). All participants who satisfy the inclusion criteria will be randomly divided into either a real mPFC-rTMS group or a sham mPFC-rTMS group in a ratio of 1:1.

#### Inclusion criteria

Participants who comply with all the following criteria will be enrolled in the clinical trial:

1. Aged 18–65
2. Meeting the diagnostic criteria for insomnia disorder according to the Diagnostic and Statistical Manual of Mental Disorders, Fifth Edition
3. PSQI score > 11 points, HAMD-14 score < 7, HAMA score < 14, and HAS score > 32
4. Sign informed consent forms for this study
5. Have not received medications for anxiety, depression, or insomnia within 4 weeks prior to enrollment in the study
6. No history of staying up late and shift in the past 4 weeks

#### Exclusion criteria

Participants who comply with one or more of the following criteria will be excluded from the clinical trial:

1. Having a family history of mental illness or other mental illness
2. A history of alcohol and drug abuse
3. Insomnia caused by other sleep disorders such as sleep apnea syndrome and restless legs syndrome
4. Serious medical diseases including the cardiovascular system, nervous system, urinary system, respiratory system, digestive system, and other serious physical diseases
5. Pregnant or breastfeeding women
6. Received physical therapy such as rTMS, transcranial direct current stimulation, or electroshock in the last months
7. Contraindication for MRI including claustrophobia, abnormal signal, or obvious asymmetrical head structure by MRI

### Oversight and dissemination

#### Composition of the coordinating center and trial steering committee

A monitoring/steering/safety committee will not be set up for this study. The study management team will be responsible for the organization of the trial including identifying potential recruits and obtaining informed consent and data collection. The study management team will meet at the beginning, the interim, and the end of the trial to monitor the study regularly to ensure adequate recruitment rate, data accuracy, and data validity.

#### Composition of the data monitoring committee, its role, and reporting structure

The study management team will be responsible for the ongoing management of the trial. In addition, we have the original medical records, and the Zhenjiang Health and Family Planning Commission will conduct a regular assessment. No data monitoring committee is needed.

#### Dissemination plans

The results of this trial will be published in a peer-reviewed journal.

#### Randomization, allocation concealment, and blinding

The eligibility participants will be randomly assigned to either a real or a sham mPFC-rTMS group with a 1:1 allocation. This study will use the Statistical Analysis Software (version 9.3, SAS Institute Inc., Cary, NC, USA) to generate the random allocation sequence. The list of randomization will be closed in computer-generated opaque envelopes with sequence numbers printed on the outside of the envelopes. After researchers screened the eligible patient, the envelopes will be opened by the researchers. The unblinded researchers will generate the allocation sequence and assign participants to interventions. The trained operators who operate rTMS will not be blinded to allocation and therefore will be excluded from assessments and data processing. Participants, statistical analysts, and assessors will be blinded. Unblinding should only be performed in case of an emergency, such as any serious adverse events.

#### Intervention

The subjects will be prohibited from receiving any other relevant treatments including drugs that can improve sleep during the trial period. *There are concomitant medications such as medications for anxiety, depression, or insomnia that are prohibited during this trial*. All relevant treatments and compliance will be recorded in the case report form. We attempted to improve adherence to interventions by strategies (i) emphasizing to participants the importance of their attendance at follow-up assessments even if they were no longer compliant with the intervention; (ii) during the follow-up period, investigators will be arranged to respond to patient inquiries; (iii) investigators will contact patients 2 (± 1) days before each visit via letters and telephone contact; and (iv) providing training in issues related to compliance for all study investigators who will contact the participants.

#### Real mPFC-rTMS group (shown in Fig. 2)

All operators who operate mPFC-rTMS will receive training to ensure consistent rTMS technique on all participants. Deep transcranial magnetic stimulation (dTMS) was administered using a Magstim Rapid2 TMS stimulator (Magstim, Whitland, UK) with H7 coil (Brainsway, Jerusalem, Israel). The front rim of the helmet was fixed at 1 cm above the nasion, then the treatment location of the H7 coil stimulates mPFC bilaterally. Before the first treatment session, single-pulse TMS was delivered to measure the resting motor threshold (RMT) of the extensor halusis brevis muscle of the participant’s toes. RMT was defined as the minimum intensity, which elicited 3 motor evoked potential (MEP) responses in 6 attempts. Treatment parameters were 1 Hz, 5-s stimulation, and 1-s stimulation intervals with a stimulation intensity of 80% of RMT. The number of repetitions was 200, the total number of pulses was 1000, and the total stimulation time was 20 min. The real mPFC-rTMS therapy will be performed 1 time per day 5 days per week for 4 weeks.

**Fig. 2:**
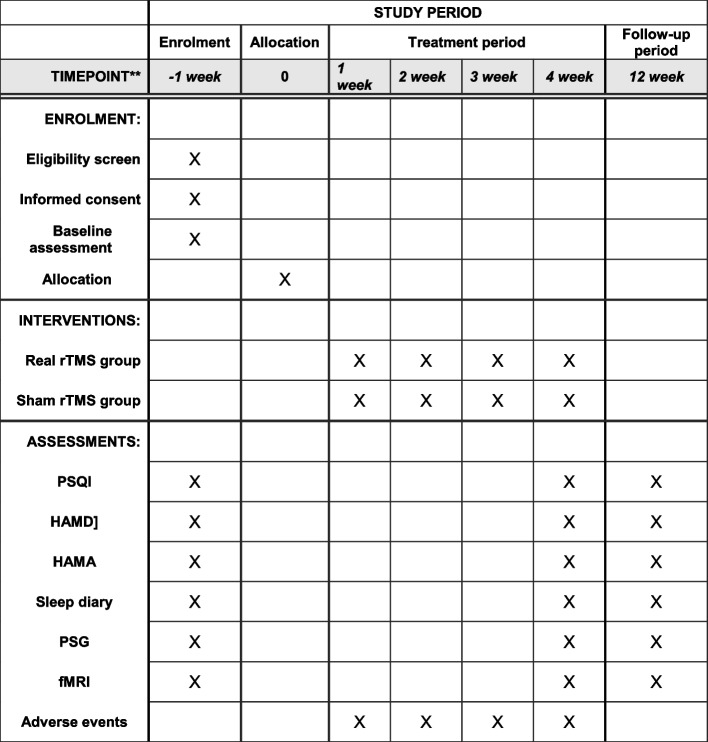
Enrollment schedule, treatment, and outcome measures. PSQI, Pittsburgh Sleep Quality Index; HAMD, Hamilton Depression Scale; HAMA, Hamilton Anxiety Scale; PSG, polysomnography; fMRI, functional magnetic resonance imaging

#### Sham mPFC-rTMS group (shown in Fig. 2)

The sham mPFC-rTMS group will be delivered by a single helmet with sham coils. The sham coil is designed to induce similar noise and scalp sensations with the same parameters as the active stimulation group.

#### Functional MRI scanning procedure

The resting-state fMRI data will be obtained at Zhenjiang Mental Health Center with a Philips 3.0-T MRI scanner. All fMRI images will be acquired from a gradient echo-echo plane imaging under the following parameters: 30 slices, repetition time (TR) = 2000 ms, echo time (TE) = 30 ms, thickness = 5 mm, field of view (FOV) =240 × 240 mm, flip angle = 90°, matrix size 64 mm × 64 mm, and total 185 volumes. Participants will be required to stay awake, keep their eyes closed, not move, close their eyes, and not try to think about anything. The real and sham mPFC-rTMS groups will be examined three times (baseline, post-treatment, and follow-up).

#### Follow-up procedure

Eight weeks after the end of treatment, the PSQI, fMRI, HAMD, HAMA, PSG, and sleep diary records will be collected.

### Outcome

#### Primary outcome

Our primary outcome will be the mean change scores of insomnia severity assessed using PSQI from baseline to the end of the treatment period. The PSQI is a 19-item insomnia assessment tool consisting of seven component scores, including sleep quality, sleep latency, sleep duration, sleep efficiency, sleep disorders, hypnotic medication, and daytime dysfunction. The higher score reflects the worse sleep quality.

#### Secondary outcomes

The secondary outcome in this study will be mean change scores of HAMD and HAMA, the weekly average of the components in the sleep diaries during the 1-week period, and changes of the sleep latency (SL), sleep efficiency (SE), total sleep time (TST), and so on which are collected through PSG between the baseline, post-treatment assessment, and 8-week follow-up.

##### Sleep diary

The sleep diary will be kept by the ID patients to record their sleeping and waking times as well as additional sleep-related factors. It is a useful tool to assess sleep quality and monitor whether treatment is working.

##### HAMD and HAMA

The HAMD and HAMA will be used to assess depression and anxiety of ID patients. In this study, we will adopt the 17-item scale of HAMD and the 14-item scale of HAMA. The HAMD and HAMA score of more than 7 points are considered as may be present depressive and anxiety symptoms.

##### PSG

We will use PSG to collect sleep parameters (TST, sleep latency, actual total sleep time, sleep efficiency) and sleep stage (N1, N2, N3, REM sleep, percentage of total sleep time in each phase).

#### Safety monitoring

Potential adverse events of mPFC-rTMS include mild headache, memory impairments, and seizures. Participants will be asked to report any adverse events at each patient visit. There is no anticipated harm and no compensation for participants.

#### Quality control and guarantee

At the begaining of the study, the whole research process, criteria for inclusion and exclusion, rTMS operation, curative effect observation, data collection and management, and adverse event reporting and recording will be trained for the researchers.

The trial will be withdrawn under the following conditions:

1. Patients with deterioration condition
2. Patients with serious adverse events who need to stop rTMS treatment
3. Other serious physical diseases during the test
4. From the medical and ethical perspective, the researcher considered that it is necessary to terminate the research
5. Participants are reluctant to continue the study and withdrawal of informed consent for non-medical reasons
6. Withdrawal of informed consent

The research data will be recorded and stored correctly, completely, and consistent with the original data in the form of double data entry under the study management team which is set up to take charge of quality control and supervise the research process. The scale evaluation is completed by designated persons. PSG is conducted by professionals in the Sleep Rehabilitation Center of Zhenjiang Mental Health Center. fMRI is conducted by professionals in the Imaging Department of Zhenjiang Mental Health Center. When the subjects drop out, the researchers will interview by face to face, phone, or mail, and so forth to ask for reasons, record the time of the last treatment, and complete the evaluation items as far as possible. Patients with deterioration conditions, adverse events, and other serious physical diseases will be taken corresponding treatment measures. Patients who have been ranked with random numbers will be included in the statistical analysis if they received more than half of the treatment course. All dropouts will be analyzed according to the intention-to-treat principle after the trial.

Patients will be numbered instead of their names. Personal information will be collected through these numbers. Informed consent forms will be secured in a locked cabinet with a special lock and a special person to keep it. Do not allow to discuss the subject’s condition or information publicly in any place in addition to the research center. We will keep patient study records confidential as relevant law which provides guarantees for the security of privacy, data, and authorized access. Any information in the study records such as name, ID number, address, and telephone number that can directly identify the patient will not be disclosed outside the Zhenjiang Mental Health Center unless required by relevant laws.

### Statistical analysis

For clinical data, statistical analyses will be analyzed using Statistical Package for the Social Sciences (version 19.0). Two-sided tests will be considered statistically significant if *P* ≤ 0.05. Cases of any missing data should be listed and the reasons should be indicated. The main efficacy analyses will be conducted based on the intention-to-treat principle. Sociodemographic information and outcome indicator will be presented with the mean ± standard deviation or the frequency (%) for the real mPFC-rTMS group and the sham mPFC-rTMS group. Independent *t* tests will be used to compare the outcome indicators between the two groups before and after mPFC-rTMS. Paired *t* tests will be used to compare the pre-rTMS and post-rTMS outcome indicators within each group. A repeated measures analysis of variance with adjustments for non-sphericity will be applied to analyze the group differences and time-dependent effects of rTMS on outcome indicators.

For fMRI data, statistical analyses will use SPM12 (http://www.fil.ion.ucl.ac.uk/spm), Data Processing & Analysis of Brain Imaging toolbox (http://rfmri.org/dpabi), and VBM8 toolbox (http://dbm.neuro.unijena.de/vbm) plugged into MATLAB_R2018a (Mathworks, Inc., Natick, MA, USA). Original data will be preprocessed: slice timing, affine head motion, nonbrain extraction, spatial smoothing, and temporal filtering will be applied. The regional resting-state fMRI time series will extract for the bilateral mPFC by using the average functional time series of all voxels within each region. Significance was set at a *P* value < 0.05 after correcting for multiple comparisons using false discovery rate (FDR) correction. Pearson correlation will be used to investigate the RSFC between bilateral mPFC and the whole-brain regions, and then a Fisher’s *r*-to-*z* transform is employed. mPFC-based functional connectivity pre-rTMS and post-rTMS between the real and sham mPFC-rTMS group will be compared using independent *t* tests. Paired *t* tests will be used to compare pre-rTMS and post-rTMS mPFC-seed functional connectivity within each group.

There will be no interim analysis.

## Discussion

Previous studies have shown that rTMS is effective in the treatment of insomnia, but no study selected targets based on neural network connectivity. We review the literature and find that mPFC may be one of the potential targets. Therefore, it is necessary to carry out further clinical trials to clarify the efficacy and possible neural mechanisms of mPFC-rTMS for ID patients. It is expected that this clinical trial will provide important data on the effect of rTMS and its possible neural mechanisms for ID.

We speculate that this effect may have relevance to the modulation mPFC-based functional connectivity. If our hypothesis is correct, functional connectivity maps of the sleep-related regions will be more modulated in the group receiving real mPFC-rTMS than in the sham mPFC-rTMS group. These results will provide insight into the potential mechanism of rTMS for ID.

## Trial status

The protocol version number is 1.1, and the date is 11 January 2020. The ethics committee of Zhenjiang Mental Health Center approved the study protocol on 3 January 2022 (permission 202007). This trial was registered on 10 December 2021 (ChiCTR2100054154). The trial started on 1 March 2022. The trial is currently recruiting participants. We predict that recruitment will be completed by 28 February 2023.

## Data Availability

All study-related data will be stored securely at the Zhenjiang Mental Health Center. The datasets analyzed during the current study are available from the corresponding author for future secondary analysis.
